# Chromosomes of *Aganaspis daci* (Weld, 1951) and a review of known karyotypes of the family Figitidae (Hymenoptera)

**DOI:** 10.3897/compcytogen.20.180400

**Published:** 2026-02-12

**Authors:** Vladimir E. Gokhman, Feliciana Pica, Fortuna Miele, Francesco Nugnes

**Affiliations:** 1 Russian Entomological Society, Moscow, Russia Russian Entomological Society Moscow Russia; 2 Institute for Sustainable Plant Protection - National Research Council (IPSP-CNR), P.le E. Fermi, 1, 80055 Portici, NA, Italy Institute for Sustainable Plant Protection - National Research Council (IPSP-CNR) Portici Italy

**Keywords:** *Aganaspis daci*, chromosome morphometry, Cynipoidea, Figitidae, karyotype, NOR, parasitoid

## Abstract

The parasitoid wasp *Aganaspis daci* (Weld, 1951) is a natural enemy of several tephritid fruit flies and was recently detected for the first time in Italy. Despite its biological and applied relevance, no chromosomal data have been available for this species until now. Here we provide the first karyotype description of *A. daci*, based on specimens from a laboratory stock in Campania (Italy), using chromosome morphometry and acridine orange staining. This species has n = 9 and 2n = 18, with a very large first metacentric chromosome, and acrocentrics prevailing among the remaining ones. The sixth metacentric presumably carries a single nucleolus organizing region (NOR). Staining with acridine orange provides homogeneous staining of all chromosomes. A brief review of the chromosome study of parasitoids of the family Figitidae, which have n = 5 to 11, is given. The haploid karyotype of ten chromosomes apparently represents the ancestral character state at least for the subfamily Eucoilinae, with chromosome sets that include n = 9 and the very large first metacentric, likely result from independent chromosomal fusions.

## Introduction

Parasitoid Hymenoptera is one of the most species-rich, taxonomically complicated, and economically important groups of insects (Bebber et al. 2014; Forbes et al. 2018). In particular, the superfamily Cynipoidea, or gallwasps, contains about 3,200 known species (Huber 2017). Moreover, Figitidae represent the most speciose family of Cynipoidea, which exceeds 1,700 described species (Ronquist 1999; Buffington et al. 2020). Although the real diversity of this group in the world fauna probably approaches 24,000 species (see Buffington et al. 2007), chromosomes of just ten members of Figitidae are known up to now (Jungen cited in Crozier 1975; Gokhman 1999, 2004; Gokhman et al. 2011, 2016; Dong et al. 2025a, b).

*Aganaspis daci* (Weld, 1951) (Hymenoptera, Figitidae, Eucoilinae) is a solitary larval-pupal endoparasitoid of several fruit fly species belonging to the genera *Anastrepha* Schiner, 1868, *Bactrocera* Macquart, 1835, and *Ceratitis* Macleay, 1829 (Diptera, Tephritidae), occurring in various subtropical regions of the world, including the Mediterranean (de Pedro et al. 2018; El-Heneidy et al. 2019; Bernardo et al. 2023).

This species was recently reported for the first time from Italy, where it was recovered from fruits infested with the Medfly *Ceratitis capitata* (Wiedemann, 1824) (Diptera: Tephritidae) in the Campania region and subsequently identified through an integrative morphological and molecular approach (Bernardo et al. 2023).

Given its potential importance as a biological control agent against tephritid pests, including *Bactrocera dorsalis* (Hendel, 1912), which has been recently detected in Italy and is currently spreading (Nugnes et al. 2018; Nugnes et al. 2024; Bernardo et al. 2025) and following previous biological and ecological studies, the present work provides the first chromosomal investigation of *A. daci* and an updated review of the known karyotypes within the family Figitidae.

## Material and methods

### Origin of the material studied

A laboratory colony of *Aganaspis daci* has been maintained at the Institute for Sustainable Plant Protection (IPSP-CNR, Portici, Italy). The colony was established in 2023 from parasitoids obtained from fruits infested in the field by *C. capitata* in the Campania region (Bernardo et al. 2023). The host, *C. capitata*, was reared according to the procedure described by Sasso et al. (2020).

The parasitoid was maintained in climate-controlled chambers at 25 ± 2 °C, 60 ± 10% relative humidity, and a 16L:8D photoperiod. Adults were kept in BugDorm cages (30 × 30 × 30 cm BioQuip products) with two mesh-covered sides for ventilation. A 50% honey solution on filter paper served as a carbohydrate source and was renewed twice per week. Part of the honey solution was also spread on the BugDorm mesh to facilitate feeding. Water was supplied ad libitum in a plastic cup closed with a perforated cap containing a sponge.

**Figure 1.**
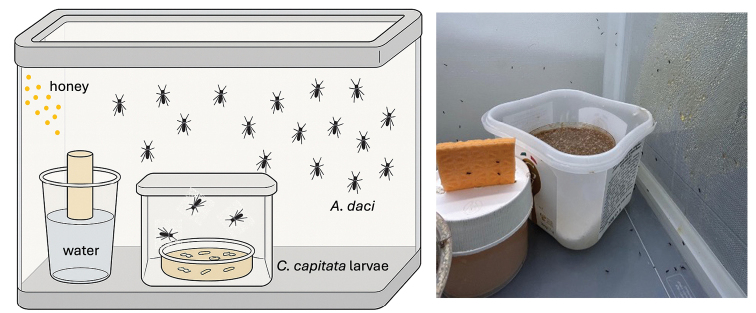
Schematic representation of the exposure setup used to obtain *Aganaspis daci* prepupae from *Ceratitis capitata* larvae reared on artificial diet (left). Close-up view of the actual rearing setup showing the cup with sponge used for water supply and the container with the Petri dish containing larvae exposed to ovipositing females of *A. daci* (right).

Second- to third-instar larvae of *C. capitata* (5–6 days old), placed in Petri dishes containing artificial diet, were exposed to ovipositing females of *A. daci*, which were allowed to parasitize the larvae. About 20 larvae were provided per female, as higher host availability promotes fecundity (de Pedro et al. 2018). Each dish was placed into 20 cm-high plastic containers to prevent larval escape and then transferred inside a BugDorm cage containing approximately 50 pairs (50 males and 50 females) of *A. daci* (Fig. 1).

Exposure lasted 24 h. This procedure was repeated daily for ten consecutive days to obtain multiple isolates for subsequent analyses. After each exposure, the larvae were transferred to Petri dishes containing fresh diet and then placed into 360- or 480-mL plastic insect jars [BRT360-BRT480 Omnes Artes (Treviglio, BG, Italy)] closed with snap-on lids fitted with fine nylon mesh to ensure adequate ventilation. Each container was labeled with both exposure and isolation dates.

Puparia of *C. capitata* parasitized by *A. daci* were used for cytogenetic analysis. Specifically, under the rearing conditions described above, the prepupal stage of *A. daci* occurred between 24 and 26 days from oviposition. This stage can be recognized by the configuration typical of the fourth larval stage (cephalic sclerites on the head capsule, the presence of several sensory structures, and two papilliform antennae) and the expulsion of meconium (Tormos et al. 2013). About 50 *C. capitata* pupae were opened at one of the two poles using minute pins, and the prepupae of *A. daci* were carefully extracted.

### Preparation and staining of chromosomes

Chromosome preparations were obtained from the cerebral ganglia of *A. daci* prepupae using a protocol broadly based on Imai et al. (1988), with minor adjustments as described in Gokhman et al. (2024).

Briefly, prepupae were dissected in the cephalic region and the cerebral ganglia were excised and immediately placed in a hypotonic solution of 0.5% sodium citrate supplemented with 0.005% colchicine.

The dissected ganglia were then transferred to a fresh aliquot of the same hypotonic solution and left to incubate for 30 min at room temperature.

Following incubation, the material was transferred onto pre-cleaned microscope slides with the aid of a Pasteur pipette. Each sample was gently rinsed on the slide with Fixative I (glacial acetic acid: absolute ethanol: distilled water, 3:3:4), and the tissue was mechanically disrupted with fine dissecting needles in an additional drop of Fixative I to obtain cell suspension.

A drop of Fixative II (glacial acetic acid: absolute ethanol, 1:1) was then applied to the same area; excess liquid was carefully wicked away from the slide margins to flatten and spread the chromosomes.

Slides were air-dried for approximately 30 min and then stored at room temperature until staining.

For routine cytogenetic examination, preparations were stained in freshly prepared 3% Giemsa solution in Sørensen’s phosphate buffer (pH = 6.8) (Sakanishi and Takayama 1977; Barcia 2007). In parallel, a subset of slides was stained with acridine orange, a DNA-binding fluorochrome, to assess possible differential fluorescence along the chromosomes. Staining followed the fluorescence-based banding procedure described by Di Berardino and Iannuzzi (1982) and subsequently employed in high-resolution karyotypic analyses (Iannuzzi et al. 1997). Specifically, the preparations were stained with 0.1% solution of acridine orange in phosphate-buffered saline (PBS, pH = 7.0) for 20 min, sequentially rinsed in tap and distilled water, and then mounted in the same buffer under sealed coverslips. Slides were examined under epifluorescence microscopy, and suitable metaphase plates were selected for imaging and morphometric analysis of chromosomes.

### Image acquisition and analysis

Metaphase plates of *A. daci* were examined and photographed using a Leica CTR 5500 microscope (Leica Microsystems, Wetzlar, Germany) equipped with a high-resolution digital camera. Image capture was performed using Leica Application Suite (LAS) software. The resulting images were processed and assembled using the image-editing program GIMP version 3.0.

KaryoType version 2.0 software (Altınordu et al. 2016) was used for taking measurements from ten haploid metaphase plates of *A. daci*. Chromosomes were classified following the guidelines provided by Levan et al. (1964).

### Data availability

All slides and data are deposited at the Institute for Sustainable Plant Protection – National Research Council (IPSP-CNR), P.le E. Fermi 1, 80055 Portici (NA), Italy, and are available upon reasonable request from Francesco Nugnes (francesco.nugnes@cnr.it).

## Results

**Figure 2.**
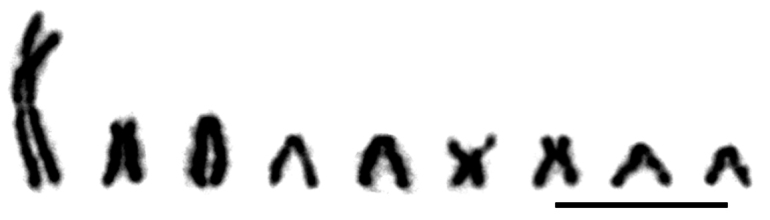
Haploid karyogram of *Aganaspis daci*. Scale bar: 10 µm.

Of the 20 slides obtained, a total of 32 haploid and 20 diploid metaphase plates from two male and four female prepupae of *Aganaspis daci* were examined, all showing the same chromosome number and morphology. The haploid karyotype consists of nine chromosomes (n = 9) (Fig. 2). Minor insignificant variation in relative lengths and centromeric indices of the chromosomes was detected among observed plates, but the overall pattern remained consistent (Table 1).

**Table 1.** Relative lengths (RLs) and centromeric indices (CIs) of chromosomes of *Aganaspis daci* (mean ± SD).

**Chromosome number**	**RL**	**CI**
1	26.82 ± 0.75	47.08 ± 2.58
2	11.76 ± 0.97	23.55 ± 3.51
3	11.41 ± 0.70	0
4	9.21 ± 0.71	0
5	8.73 ± 0.36	0
6	8.70 ± 0.45	42.56 ± 3.29
7	8.62 ± 0.75	36.01 ± 3.15
8	8.02 ± 0.38	0
9	6.73 ± 0.42	0

Chromosome 1 is a very large metacentric (centromeric index, CI = 47.08 ± 2.58), accounting on average for ~27% of the total haploid set (relative length, RL = 26.82 ± 0.75%). This element is more than twice as long as any of the remaining chromosomes, making it a clear marker of the karyotype.

Chromosomes 2 and 3 are of comparable size (RL = 11.76 ± 0.97% and 11.41 ± 0.70%, respectively), but differ in morphology: chromosome 2 shows an asymmetric centromere position (CI = 23.55 ± 3.51), corresponding to a submetacentric to subtelocentric morphology, whereas chromosome 3 is acrocentric (CI = 0). The medium-sized chromosomes (4–8) range between ~8% and ~9% RL. Chromosomes 4, 5, 8 and 9 are acrocentric (CI = 0), with chromosome 9 representing the smallest element in the set (RL = 6.73 ± 0.42%). Chromosome 6 is metacentric (CI = 42.56 ± 3.29) and chromosome 7 is metacentric to submetacentric (CI = 36.01 ± 3.15). The diploid chromosome number of *A. daci* is therefore 2n = 18.

Chromosome 6 carries a distinct secondary constriction and small terminal satellites on its short arm (Fig. 2). Such satellites are typically associated with nucleolus organizing regions (NORs), suggesting that this element harbors the main rDNA locus in this species.

**Figure 3.**
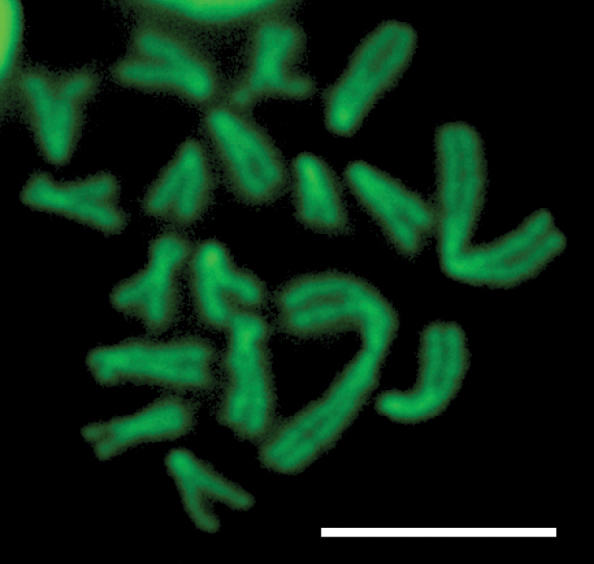
Diploid metaphase plate of *Aganaspis daci* stained with acridine orange. Scale bar: 10 µm.

Fluorescent staining with acridine orange resulted in homogeneous labeling of the chromosome set and did not reveal detectable banding differentiation among or within chromosomes (Fig. 3). These results apparently confirm that the chromosomal preparation technique used for karyotyping *A. daci* adequately captured the native structure of chromosomes in this species. In addition, no heteromorphic regions or structural variants were observed in the examined plates.

## Discussion

Haploid chromosome numbers in the family Figitidae vary from 5 to 11, with most karyotypes having n = 9–10 (Table 2). In *Aganaspis daci*, the haploid and diploid number of nine and eighteen chromosomes respectively and the absence of any intraspecific variation in morphology or number indicate a stable and well-defined karyotype. This chromosomal stability may reflect a conserved genomic architecture within the species, providing a reliable basis for comparative and evolutionary analyses.

**Table 2.** Chromosome numbers of parasitoids of the family Figitidae. *Results of chromosome-level genome assemblies.

**Species**	**n(2n)**	**Reference**
Subfamily Charipinae		
*Phaenoglyphis villosa* (Hartig, 1841)	(20)	Gokhman 2004
Subfamily Aspicerinae		
*Callaspidia defonscolombei* Dahlbom, 1842	11	Gokhman 1999
Subfamily Eucoilinae		
*Aganaspis daci* (Weld, 1951)	9(18)	Present paper
*Ganaspis xanthopoda* (Ashmead, 1896)	9	Gokhman et al. 2011
*Leptopilina boulardi* (Barbotin et al., 1979)	9(18)	Gokhman et al. 2011; Dong et al. 2025a*
*Leptopilina clavipes* (Hartig, 1841)	5	Pannebakker et al. 2004
*Leptopilina drosophilae* (Kieffer, 1913)	10	Dong et al. 2025a*
*Leptopilina heterotoma* (Thomson, 1862)	10(20)	Jungen cited in Crozier 1975; Gokhman et al. 2011; Dong et al. 2025a*
*Leptopilina myrica* Chen, 2023	10(20)	Dong et al. 2025a*, b
*Leptopilina syphax* Quinlan, 1988	10	Dong et al. 2025a*
*Leptopilina victoriae* Nordlander, 1980	10	Gokhman et al. 2011

In the family Figitidae, metacentric and submetacentric chromosomes are generally predominant, although subtelocentric and acrocentric elements also occur in many species’ karyotypes (Gokhman 2004; Gokhman et al. 2011; Dong et al. 2025b).

Although chromosome sets have been studied in only eleven species of Figitidae so far, certain patterns of karyotype evolution can already be outlined, particularly within the subfamily Eucoilinae. Specifically, the most frequent haploid number n = 10 most likely represents the ancestral chromosome number for this subfamily, and probably also for the family as a whole (see Buffington et al. 2007).

If this assumption is correct, then karyotypes with n = 9 [*A. daci*, *Ganaspis xanthopoda* (Ashmead, 1896), *Leptopilina boulardi* (Barbotin et al., 1979)] and n = 5 [*Leptopilina clavipes* (Hartig, 1841)] are apparently derived.

Notably, the chromosome sets of the first three species contain a very large metacentric chromosome, suggesting that chromosomal fusions have occurred in karyotypes originally bearing n = 10, similarly to gall wasps of the genus *Isocolus* Förster, 1869 (Cynipidae) (Gokhman 2021). At least in the case of *L. boulardi*, fusion of this kind is also confirmed by the results of chromosome-level genome assembly (Dong et al. 2025a). Because *A. daci*, *G. xanthopoda*, and *L. boulardi* belong to three distantly related genera, the above-mentioned fusions most likely took place independently (see Buffington et al. 2007). This interpretation is further supported by the differing positions of the nucleolus organizing regions (NORs), which correspond to the ribosomal DNA (rDNA) loci in various members of Figitidae (Gokhman et al. 2016; present study; see also Gokhman and Kuznetsova 2024).

Moreover, at least both *A. daci* and *L. boulardi* possess a relatively higher proportion of subtelocentric and acrocentric chromosomes. Combined with the smaller chromosome size and lower genome size of the latter species, this pattern may reflect deletions of constitutive heterochromatin (Gokhman et al. 2011). If this is the case, the mechanisms responsible for changes in chromosome number in *Leptopilina* Förster, 1869 (with n = 5, 9, 10) resemble those described in certain genera of chalcid wasps such as *Eurytoma* Illiger, 1807 and *Sycophila* Walker, 1871 (Eurytomidae) as well as *Metaphycus* Mercet, 1917 (Encyrtidae), where n likewise varies between 5 and 10 (Gokhman 2020, 2022). In addition, the results of chromosome-level genome assemblies of several *Leptopilina* species (Dong et al. 2025a) suggest that certain inversions are involved in the process of karyotype evolution within this genus.

At present, no karyotypically distinct cryptic species of Figitidae have been reported. However, this situation may change as more species are examined, as has occurred in other groups of parasitic Hymenoptera, e.g., Cynipidae (Abe 1998, 2007; Gokhman 2009, 2022). Further cytogenetic investigation within Figitidae will likely yield valuable insights into their genetic architecture and may provide information of practical relevance for the mass rearing and applied use of these parasitoids.

## Conclusion

This study provides the first karyotype description of *Aganaspis daci*, revealing a haploid set of nine chromosomes with a very large metacentric element. Comparative analysis suggests that chromosome fusions, together with inversions and changes in the amount of constitutive heterochromatin, played a substantial role in the karyotype evolution of the Figitidae, furthermore contributing to the understanding of chromosomal diversity in parasitoid wasps.

## References

[B1] Abe Y (1998) Karyotype differences and speciation in the gall wasp *Andricus mukaigawae* (s. lat.) (Hymenoptera: Cynipidae), with description of the new species *A. kashiwaphilus*. Entomologica Scandinavica 29: 131–135. 10.1163/187631298X00249

[B2] Abe Y (2007) Parallelism in secondary loss of sex from a heterogonic life cycle on different host plants in the *Andricus mukaigawae* complex (Hymenoptera: Cynipidae), with taxonomic notes. Journal of Natural History 41: 473–480. 10.1080/00222930701192122

[B3] Altınordu F, Peruzzi L, Yu Y, He X (2016) A tool for the analysis of chromosomes: KaryoType. Taxon 65(3): 586–592. 10.12705/653.9

[B4] Barcia JJ (2007) The Giemsa stain: Its history and applications. International Journal of Surgical Pathology 15(3): 292–296. 10.1177/1066896907302239 17652540

[B5] Bebber DP, Polaszek A, Wood JRI, Barker C, Scotland RW (2014) Taxonomic capacity and author inflation. New Phytologist 202: 741–742. 10.1111/nph.12745 24716516

[B6] Bernardo U, Pica F, Carbone C, Nugnes F, Viggiani G (2023) First record and characterization of *Aganaspis daci* (Weld, 1951) (Hymenoptera, Figitidae, Eucoilinae), a parasitoid of fruit flies, from Italy. Journal of Hymenoptera Research 96: 863–877. 10.3897/jhr.96.110000

[B7] Bernardo U, Nugnes F, Ascolese R, Miele F, Innangi M, Di Febbraro M (2025) Predicting the invasion risk of *Bactrocera dorsalis* in Italy under climate and land cover change. Scientific Reports 15: 35096. 10.1038/s41598-025-18890-2 PMC12508463 41062602

[B8] Buffington ML, Nylander JAA, Heraty JM (2007) The phylogeny and evolution of Figitidae (Hymenoptera: Cynipoidea). Cladistics 23: 403–431. 10.1111/j.1096-0031.2007.00153.x

[B9] Buffington ML, Forshage M, Liljeblad J, Tang CT, van Noort S (2020) World Cynipoidea (Hymenoptera): A key to higher-level groups. Insect Systematics and Diversity 4(1): 1. 10.1093/isd/ixaa003

[B10] Crozier RH (1975) Animal Cytogenetics 3(7). Gebrüder Borntraeger, Berlin–Stuttgart, 95 pp.

[B11] de Pedro L, Tormos J, Asís JD, Sabater-Muñoz B, Beitia F (2018) Biology of *Aganaspis daci* (Hymenoptera: Figitidae), parasitoid of *Ceratitis capitata* (Diptera: Tephritidae): Mode of reproduction, biological parameters and superparasitism. Crop Protection 108: 54–61. 10.1016/j.cropro.2018.02.015

[B12] Di Berardino D, Iannuzzi L (1982) Detailed description of R-banded bovine chromosomes. Journal of Heredity 73(6): 434–438. 10.1093/oxfordjournals.jhered.a109693 7153495

[B13] Dong Z, Lu Y, Fang G, Zhang Q, Sheng Y, Pang L, Chen J, Shi W, Feng T, Zhang J, Li G, Chen X, Huang J, Zhan S (2025a) A rare, evolutionarily conserved venom protein benefits endoparasitism across parasitoids. Cell Genomics 5: 100920. 10.1016/j.xgen.2025.100920 PMC12366661 40541180

[B14] Dong Z, Xu Z, Zhang J, Guo Y, Zhang Q, Pang L, Feng T, Shi W, Sheng Y, Huang J, Chen J (2025b) Chromosome-level genome sequencing and assembly of the parasitoid wasp *Leptopilina myrica*. Scientific Data 12: 235. 10.1038/s41597-025-04577-w PMC11807108 39922808

[B15] El-Heneidy AH, Hosni ME, Ramadan MM (2019) Identity and biology of *Aganaspis daci* (Weld) (Hymenoptera: Figitidae), recently introduced to Egypt for biological control of *Bactrocera zonata* (Diptera: Tephritidae). Entomologist’s Monthly Magazine 155(1): 17–37. 10.31184/m00138908.1551.3958

[B16] Forbes AA, Bagley RK, Beer MA, Hippee AC, Widmayer HA (2018) Quantifying the unquantifiable: Why Hymenoptera, not Coleoptera, is the most speciose animal order. BMC Ecology 18: 21. 10.1186/s12898-018-0176-x PMC6042248 30001194

[B17] Gokhman VE (1999) Chromosomes of *Callaspidia defonscolombei* (Hymenoptera, Figitidae). Zoologichesky Zhurnal 78: 1476–1477. [In Russian]

[B18] Gokhman VE (2004) Chromosomes of *Phaenoglyphis villosa* (Hartig, 1841) (Hymenoptera: Figitidae). Russian Entomological Journal 13(4): 267–268.

[B19] Gokhman VE (2009) Karyotypes of Parasitic Hymenoptera. Springer, Dordrecht, 183 pp. 10.1007/978-1-4020-9807-9

[B20] Gokhman VE (2020) Chromosomes of parasitic wasps of the superfamily Chalcidoidea (Hymenoptera): An overview. Comparative Cytogenetics 14(3): 399–416. 10.3897/compcytogen.v14i3.56535 PMC9849058 36761105

[B21] Gokhman VE (2021) Chromosomes of three gall wasps of the tribe Aylacini (Hymenoptera, Cynipidae). Comparative Cytogenetics 15(2): 171–178. 10.3897/compcytogen.v15.i2.66781 PMC8195943 34131479

[B22] Gokhman VE (2022) Comparative karyotype analysis of parasitoid Hymenoptera (Insecta): major approaches, techniques, and results. Genes 13: 751. 10.3390/genes13050751 PMC9141968 35627136

[B23] Gokhman VE, Kuznetsova VG (2024) Structure and evolution of ribosomal genes of insect chromosomes. Insects 15: 593. 10.3390/insects15080593 PMC11354594 39194798

[B24] Gokhman VE, Johnston JS, Small C, Rajwani R, Hanrahan SJ, Govind S (2011) Genomic and karyotypic variation in *Drosophila* parasitoids (Hymenoptera, Cynipoidea, Figitidae). Comparative Cytogenetics 5(3): 211–221. 10.3897/compcytogen.v5i3.1435 PMC3833773 24260630

[B25] Gokhman VE, Bolsheva NL, Govind S, Muravenko OV (2016) A comparative cytogenetic study of *Drosophila* parasitoids (Hymenoptera, Figitidae) using DNA-binding fluorochromes and FISH with 45S rDNA probe. Genetica 144: 335–339. 10.1007/s10709-016-9902-5 27150102

[B26] Gokhman VE, Luna MG, Vallina C, Bressa MJ (2024) Chromosomes of *Pseudapanteles dignus* (Muesebeck, 1938) and a review of known karyotypes of the subfamily Microgastrinae (Hymenoptera, Braconidae). Comparative Cytogenetics 18: 199–211. 10.3897/compcytogen.18.133534 PMC11605297 39619648

[B27] Huber JT (2017) Biodiversity of Hymenoptera. In: Foottit RG, Adler PH (Eds) Insect Biodiversity: Science and Society. 2^nd^ ed. Wiley Blackwell, Oxford, 419–461. 10.1002/9781118945568.ch12

[B28] Iannuzzi L, Di Meo GP, Perucatti A (1997) The high-resolution GBA + CBA-banded karyotype in cattle (*Bos taurus* L.). Caryologia 50: 59–66. 10.1080/00087114.1997.10797384

[B29] Imai HT, Taylor RW, Crosland MWJ, Crozier RH (1988) Modes of spontaneous chromosomal mutation and karyotype evolution in ants with reference to the minimum interaction hypothesis. Japanese Journal of Genetics 63: 159–185. 10.1266/jjg.63.159 3273765

[B30] Levan A, Fredga K, Sandberg AA (1964) Nomenclature for centromeric position on chromosomes. Hereditas 52: 201–220. 10.1111/j.1601-5223.1964.tb01953.x

[B31] Nugnes F, Russo E, Viggiani G, Bernardo U (2018) First record of an invasive fruit fly belonging to *Bactrocera dorsalis* complex (Diptera: Tephritidae) in Europe. Insects 9(4): 182. 10.3390/insects9040182 PMC6316371 30513969

[B32] Nugnes F, Carbone C, Ascolese R, Miele F, Pica F, Palmieri A, Griffo R, Bernardo U (2024) The enemy is already inside! *Bactrocera dorsalis* is a serious threat to European orchards and crops. Entomologia Generalis 44: 1243–1251. 10.1127/entomologia/2024/2613

[B33] Pannebakker BA, Pijnacker LP, Zwaan BJ, Beukeboom LW (2004) Cytology of *Wolbachia*-induced parthenogenesis in *Leptopilina clavipes* (Hymenoptera: Figitidae). Genome 47: 299–303. 10.1139/g03-137 15060582

[B34] Ronquist F (1999) Phylogeny, classification and evolution of the Cynipoidea. Zoologica Scripta 28: 139–164. 10.1046/j.1463-6409.1999.00022.x

[B35] Sakanishi S, Takayama S (1977) A simple Giemsa method for the differential staining of sister chromatids with a note on the presumptive mechanism involved. Proceedings of the Japan Academy, Series B 53: 143–146. 10.2183/pjab.53.143

[B36] Sasso R, Gualtieri L, Russo E, Nugnes F, Gebiola M, Bernardo U (2020) The establishment of a rearing technique for the fruit fly parasitoid *Baryscapus silvestrii* increases knowledge of biological, ecological and behavioural traits. BioControl 65(1): 47–57. 10.1007/s10526-019-09984-8

[B37] Tormos J, De Pedro L, Beitia F, Sabater B, Asís JD, Polidori C (2013) Development, preimaginal phases and adult sensillar equipment in *Aganaspis* parasitoids (Hymenoptera: Figitidae) of fruit flies. Microscopy and Microanalysis 19: 1475–1489. 10.1017/S1431927613013330 23985273

